# Integrating animal tracking and trait data to facilitate global ecological discoveries

**DOI:** 10.1242/jeb.247981

**Published:** 2025-02-20

**Authors:** Roxanne S. Beltran, A. Marm Kilpatrick, Stephanie K. Adamczak, Larissa T. Beumer, Max F. Czapanskiy, Sarah C. Davidson, Bryan S. McLean, Thomas Mueller, Allison R. Payne, Carmen D. Soria, Brian C. Weeks, Terrie M. Williams, Roberto Salguero-Gómez

**Affiliations:** ^1^ Department of Ecology and Evolutionary Biology, University of California Santa Cruz, Santa Cruz, CA 95064, USA; ^2^ The University Centre in Svalbard, Longyearbyen 9170, Svalbard, Norway; ^3^ Institute of Marine Sciences, University of California Santa Cruz, Santa Cruz, CA 95064, USA; ^4^ Department of Migration, Max Planck Institute of Animal Behavior, 78315 Radolfzell, Konstanz, Germany; ^5^ Department of Biology, University of Konstanz, 78464 Konstanz, Germany; ^6^ Department of Biology, University of North Carolina at Greensboro, Greensboro, NC 27412, USA; ^7^ Senckenberg Biodiversity and Climate Research Centre (SBiK-F), 60325 Frankfurt am Main, Germany; ^8^ Department of Biological Sciences, Goethe University, 60323 Frankfurt am Main, Germany; ^9^ Department of Spatial Sciences, Faculty of Environmental Sciences, Czech University of Life Sciences Prague, 165 00 Praha-Suchdol, Czech Republic; ^10^ School for Environment and Sustainability, University of Michigan, Ann Arbor, MI 48109, USA; ^11^ Department of Biology, University of Oxford, Oxford OX1 3RB, UK

**Keywords:** Biologging, Integration, Macroecology, Repository, Tracking data, Trait data

## Abstract

Understanding animal movement is at the core of ecology, evolution and conservation science. Big data approaches for animal tracking have facilitated impactful synthesis research on spatial biology and behavior in ecologically important and human-impacted regions. Similarly, databases of animal traits (e.g. body size, limb length, locomotion method, lifespan) have been used for a wide range of comparative questions, with emerging data being shared at the level of individuals and populations. Here, we argue that the proliferation of both types of publicly available data creates exciting opportunities to unlock new avenues of research, such as spatial planning and ecological forecasting. We assessed the feasibility of combining animal tracking and trait databases to develop and test hypotheses across geographic, temporal and biological allometric scales. We identified multiple research questions addressing performance and distribution constraints that could be answered by integrating trait and tracking data. For example, how do physiological (e.g. metabolic rates) and biomechanical traits (e.g. limb length, locomotion form) influence migration distances? We illustrate the potential of our framework with three case studies that effectively integrate trait and tracking data for comparative research. An important challenge ahead is the lack of taxonomic and spatial overlap in trait and tracking databases. We identify critical next steps for future integration of tracking and trait databases, with the most impactful being open and interlinked individual-level data. Coordinated efforts to combine trait and tracking databases will accelerate global ecological and evolutionary insights and inform conservation and management decisions in our changing world.

## Teaser

**Summary:** We believe that coordinated efforts to combine trait and tracking databases will accelerate global ecological and evolutionary insights and inform conservation and management decisions in our changing world.

## Introduction

Understanding the feedback loops between animal attributes and their movements can catalyze the search for general laws in ecology, evolutionary and conservation biology (Nathan et al., 2008; Sutherland et al., 2013). For example, how does body size relate to maximum migration distances of species that walk, swim or fly? How do size, sex and reproductive strategies modulate movement patterns? Macroecology has relied on cross-species comparative analyses to address these questions at a global scale (Pottier et al., 2024). More recently, advancements in biologging technologies (i.e. instruments attached to animals to monitor their behavior and physiology) have separately provided unprecedented opportunities for research across biological scales, from individuals to ecosystems (Williams et al., 2020). Here, we propose that the integration of functional eco-physiology and movement ecology offers a powerful framework towards fundamental and applied research regarding the drivers and consequences of vertebrate movement across scales. We focus on mammals and birds, because most datasets currently available in open-access repositories are richest for these taxa (Conde et al., 2019; Troudet et al., 2017).

## Trait databases

Organisms can be described by a suite of traits, i.e. morphological, physiological, phenological, behavioral and life history characteristics, that influence their performance and ecosystem effects (Dawson et al., 2021; Streit and Bellwood, 2023) (Box 1). Organismal traits are often measured on museum specimens or in field studies, and collections of these traits are published, as manuscripts with supplemental tables (e.g. McNab, 2008; Tobias et al., 2022; Tombak et al., 2024), stand-alone databases (e.g. Gainsbury et al., 2018; Faurby et al., 2018; Jones et al., 2009) or ‘meta-databases’ (i.e. databases of databases, e.g. Soria et al., 2021; Balk et al., 2022; Myhrvold et al., 2015). For example, COMBINE (a COalesced Mammal dataBase of INtrinsic and Extrinsic traits; Soria et al., 2021) contains information on 54 traits for 6234 extant and recently extinct mammal species, including information on morphology, reproduction, diet, life habit, phenology, behavior and home range. Most of these databases contain data at the species level, typically presented as mean, minimum, maximum or range of values derived from several individuals or studies, with limited information on intraspecific variation or sample sizes. However, databases such as iDigBio, VertNet and FuTRES – which are collated exclusively from museum specimens – contain traits linked to individuals (Balk et al., 2022). Trait databases such as these are enabling researchers to address a vast array of questions at granular scales. For example, intraspecific studies have provided insight into macro-scale spatiotemporal patterns of variation in body size (Hantak et al., 2021) and breeding phenology (McLean et al., 2022; McLean and Guralnick, 2021). Likewise, interspecific studies have discovered the drivers of litter size (Weller et al., 2024), lifespan (Healy et al., 2014), urban tolerance (Neate-Clegg et al., 2023) and rates of contemporary change (Zimova et al., 2023), and underscored the importance of continuous multivariate trait data at large scales (Weeks et al., 2022). Some meta-analyses have linked traits including physiological parameters with behavioral states and activity levels (Wu and Seebacher, 2022; Mathot et al., 2019; Arnold et al., 2021), but movement data are often lacking from these macroecological studies.

**Box 1. Questions that could be answered by integrating information from tracking and trait databases**

Tracking and trait databases contain extensive information about global patterns (see figure); their integration could enable researchers to answer key questions about ecological and evolutionary processes. Note that some metrics (e.g. demographic traits) can be represented in both trait (e.g. lifespan) and tracking (e.g. mortality signal) databases. The sorts of questions that might be addressed by referencing tracking and trait databases are as follows:

• How do responses to climate change (timing or spatial extent of movement) relate to traits (reproductive strategy, diet specialization, morphology, transport modality, thermal tolerance, breeding strategy?

• How do responses to climate change (indexed by northern range bounds across years) relate to body size?

• How do physiological and biomechanical traits (metabolic rates, leg length, wingspan) relate to migration distances?

• How do species’ body size and performance limits (maximum transit speed) constrain life history timing in movement/migration?

• Which traits drive an individual's propensity to migrate in partially migrating species?

• How do traits (body size, sex) contribute to utilization of specifi­c migration routes or conservation corridors?

• How does brain size relate to the degree of phenological compensation (moderating speed based on distance)?

• How does the cost per step/stroke relate to body size, transport modality and movement distance?

• How does litter size and frequency relate to phenology for migrating species?

• How does gestation duration relate to migration distance/location/direction?

• Do ‘lucky’ foraging strategies (feeding rate variance) slow down the pace of life history (longevity, age at maturity, reproductive rate)?

• How do migration distance and migration duration influence lifespan and lifetime reproductive output?

• How do movement distance, metabolic rate and cost per step/stroke scale up to total migration costs?

• How do experimentally induced changes in physiology impact the timing of life history events, including migration?

• How do traits (body size, limb characteristics, diet specialization, nocturnality/diurnality) relate to home range size and daily movements?

• Do partially migrating species have different traits to obligate migrators?

• How does phenotypic plasticity in metabolically related traits (body size, skeletal proportions, organ size) change daily movements and home range across seasons?
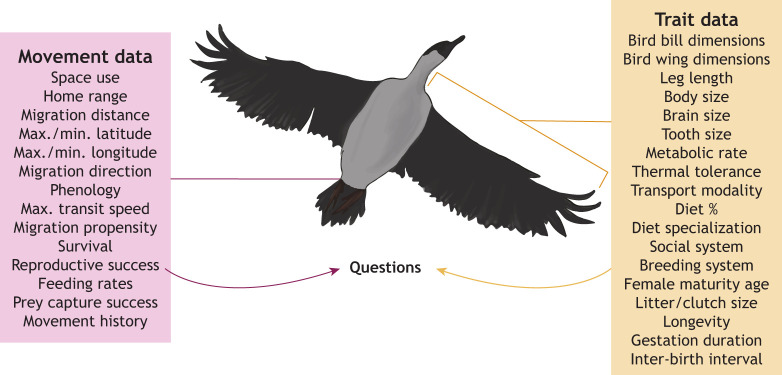

## Tracking databases

A key trait of animals is their mobility, which drastically extends and shapes their interactions with the abiotic and biotic environment (Bryce et al., 2022; Abrahms et al., 2021). Biologging and biotelemetry techniques – the use of animal-borne sensors to monitor location, behavior and performance over time and space (Watanabe and Papastamatiou, 2023; Cooke et al., 2004; Rutz and Hays, 2009) – are central to the field of movement ecology (Nathan et al., 2008). Summaries of tracking data are often published in supplemental tables (Joly et al., 2019) and, increasingly, raw data are made available in data papers (Ropert-Coudert et al., 2020) or databases (Kranstauber et al., 2011). For example, Movebank (Kays et al., 2022) is a global data platform for animal tracking that (as of December 2024) contains 7.5 billion location records, including 8843 studies and 1478 taxa. All data in Movebank are harmonized to a shared data model and vocabulary (http://vocab.nerc.ac.uk/collection/MVB/current/). This vocabulary stores individual location and sensor measurement records, including a time stamp and associated animal and device identifiers, which can be used to calculate many key movement metrics (Box 1). These data expand our knowledge beyond geographic distribution (range/occurrence) by including temporally explicit movement information that describes different types of movements (i.e. natal dispersal, seasonal migrations, daily movements). Further, Movebank supports storage of trait measurements commonly taken at the time of device attachment or retrieval. Tracking databases have allowed for a suite of impactful research papers across disciplines and levels of biological organization (Davidson et al., 2020; Hindell et al., 2020; Abrahms et al., 2017; Beltran et al., 2024). Although multiple tracking databases exist (many of which integrate trait data), here we focus on Movebank because of its global coverage, broad usage by researchers and practitioners, and Application Programming Interface enabling efficient extraction of the public trait data we assessed.

## Interoperability between trait and tracking databases

Although trait and tracking databases have both matured rapidly in recent years, they are rarely combined for macroecology research. The harmonization of tracking and trait databases could be part of a much broader shift towards the full interoperability of databases in ecology (Gallagher et al., 2020). Recent efforts in this direction have resulted in the examination of the influence of body size on maximum migration distance and displacement from human disturbance (Hein et al., 2012; Doherty et al., 2021). Other work has suggested that trophic guild likely mediates global reductions in mammal movements in high human density areas (Tucker et al., 2018). Bird banding data have been used to link natal dispersal with wing shape, including wing aspect ratio (Claramunt, 2021) and hand-wing index (Weeks et al., 2022), and the hand-wing index has been used to address questions regarding the ecological processes linked to dispersal ability variation at a global scale (Sheard et al., 2020). Although banding data contain valuable individual-level movement data, often paired with measured traits, here we focus instead on tracking data because they provide valuable information on emigration and mortality events that are not captured in banding data.

The integration of tracking data with trait data could enhance the generality of research in ecology and evolution in mammals and birds (Box 1). However, this vision will require overcoming some key challenges associated with long-term, individual-level ecological datasets, including harmonization of datasets, their own biases, and the expertise of researchers who develop and curate them (Salguero-Gómez et al., 2021). Here, we detail these challenges and ways to address them.

### Disparities in data-sharing norms

Trait data repositories commonly adopt open science principles (Gallagher et al., 2020), whereas sharing in tracking data repositories has historically been more limited (Campbell et al., 2016; Sequeira et al., 2021; Davidson et al., 2025). Making trait data publicly available is becoming the norm (Gallagher et al., 2020), possibly because trait-based analyses rely so much on synthesis and communal resources such as museum collections (where reporting catalog numbers alongside derivative data is the norm in publication and often a precondition of using collections) or databases generated using decades of public investment (e.g. long-term banding operations). As a result, researchers who publish trait measurements often provide full access to the data. We are not aware of an assessment of the proportion of collected trait data that are published or contributed to open trait databases. In contrast, while there is no available estimate of the number of animals equipped with tracking devices globally (Rutz, 2022), only a portion of tracking data are publicly discoverable or available to date (Campbell et al., 2015). Support for non-public tracking data is a prerequisite for databases and collaborative projects to enhance the use of data on endangered species, real-time and legally restricted data, government data that require agency sharing agreements for use, and data collection for ongoing studies that have not yet been published.

To better understand disparities in data-sharing norms, we accessed trait databases that contain data from only birds [AVONET (Tobias et al., 2022)], only mammals [PHYLACINE (Faurby et al., 2018), PanTHERIA (Jones et al., 2009)] and both birds and mammals [AnAge (Tacutu et al., 2013; De Magalhães and Costa, 2009), EltonTraits (Wilman et al., 2014)] along with tracking data from both birds and mammals [Movebank (Kays et al., 2022)] (Table S1). Specifically, we extracted genus, species and adult body size information from all databases. These trait databases are designed to provide open access to collected data, whereas Movebank, like most tracking databases, offers a suite of services including open and controlled data sharing. In Movebank (Kays et al., 2022), likely the largest tracking database, at the time of access (March 2024), 13% (744 out of 5659) of studies were available for open access download, of which 11% (616 species) corresponded to mammals and birds (with license types CC_BY=178, CC_BY_NC=96, CC_0=245, CUSTOM=93) (Table S2). These 616 studies contained data from 28,105 individual tracking data deployments.

Many biologging repositories are designed for collaborative data collection [Ocean Tracking Network (Iverson et al., 2018)] or research syntheses [EuroMammals (Urbano et al., 2021)], often with flexible sharing requirements to promote participation. However, the lack of mandates on data publication also presents challenges for data persistence and replicability. We argue that there are two possible reasons for this difference in current data-sharing norms between trait and tracking data: the inevitable costs of collecting tracking data, and conservation concerns associated with publicly available tracking data. The first of these includes the financial costs and time investments to individual researchers who collect and curate animal tracking data. Tracking data costs are substantially higher (hundreds to thousands of dollars per animal in instruments) relative to the cost of measuring animal traits from specimens that have already been collected and curated (tens to hundreds of dollars per animal in equipment). Moreover, the costs of tracking data are proportionally higher relative to resource availability in nations with limited research funding. The second reason for the lower accessibility of tracking data relative to trait data is the conservation concerns related to high-resolution tracking data of endangered species, especially those with high site fidelity. One solution to the limited access to tracking data is for funding agencies and journals to continue mandating open access to data, as has been done with the specimens and other samples (Colella et al., 2021), but such mandates should carefully consider equity and conservation concerns (Colella et al., 2021), including specific attribute fields (Gascoigne et al., 2023; Wieczorek et al., 2012). A potential alternative is for these entities to mandate discoverability of studies so data owners could be contacted to discuss potential use. In addition, clear ownership and license agreements can maximize the likelihood that proper credit is given when the data are used. For example, Movebank offers a public data repository that supports dataset curation, citation licenses and persistent identifiers (DOIs) (Kays et al., 2022), and government agencies have designed similar archiving efforts using Movebank's data format (e.g. van der Kolk et al., 2022). Likewise, tracking databases could develop a community-endorsed set of data standards to adopt, like those common in trait databases (Schneider et al., 2019).

### Disconnections in taxonomic keys

Another consideration for merging trait and tracking data is finding matching taxonomic keys (e.g. common name, Latin name) across databases (Grenié et al., 2023). Users can take advantage of the existing wide functionality [e.g. the R package *taxize* (Chamberlain and Szöcs, 2013)] to search over various taxonomic data sources such as the Integrated Taxonomic Information System (ITIS) for scientific and common species names, along with upstream taxonomic classifications (e.g. family, order). Taxonomy crosswalks, or tables that show equivalent fields in multiple database schema, can also be used to connect the data across different datasets [e.g. phylogeny and range maps (Tobias et al., 2022), or in our case, trait and tracking data]. All databases that we examined had genus and species formatted in a consistent manner, which allowed them to be used as the merging key. It will also be important to establish norms in reporting which version of taxonomies have been used to label trait and tracking data, so that taxonomic changes can be accounted for over time.

### Disconnections among levels of organization

Data format and complexity differ substantially between trait and tracking databases. In their simplest form, trait databases associate trait values with an individual (e.g. Balk et al., 2022), population (e.g. Levin et al., 2022; Salguero-Gómez et al., 2016; Santini et al., 2018) or species (e.g. Wilman et al., 2014). Moreover, trait databases often exist in a more readily usable form than spatiotemporal data from biologging sensors in tracking databases, which must be processed to obtain the metric of interest (e.g. migration distance). Another major challenge of integrating trait and tracking databases is that most trait databases still hold data at the species level (often without calculation methods, variance measures or sample sizes), whereas tracking databases hold data at the individual level. One possible solution for synthesis would be to aggregate tracking data down to the species level and include associated sample size and uncertainty. However, trait-based datasets should also consider reporting information at the individual level, as substantial intraspecific variation exists in species traits (Zheng et al., 2023). A key benefit of aggregation is being able to match tracking data with trait data for the many species that are only available at the species level. However, aggregation of tracking data can obscure important intraspecific variation. An alternative to aggregation that may become more tenable soon is using tracking data at the individual level and pairing it with the more limited, but growing, individual-level trait databases. This approach would make it possible to calculate covariation between traits and tracking. Doing so substantially reduces available data in which tracking data are accompanied by individual-level trait data (e.g. age, sex, body size); for example, only 24% of tracking datasets from Movebank were associated with individual trait data in our data download. Additionally, it is often difficult to measure many individual-level traits while instrumenting living animals (i.e. during physical or chemical restraint) in the same way that non-living animals can be measured. While advanced methods are facilitating increased intra-specific trait sampling from museum specimens (e.g. Weeks et al., 2023), overlapping tracking and trait data are still elusive because the specimens are unlikely to have historical tracking data, and resources to link representations of individuals across data platforms are still in early stages (Hardisty et al., 2022). Therefore, for the foreseeable future, the most likely path forward may be to generate and deposit individual-level trait data. We also expect that the emerging application of expert-trained artificial intelligence to biological images such as camera trap photos will become more useful in generating estimates of trait values.

### Disparities across the tree of life

**Fig. 1.**
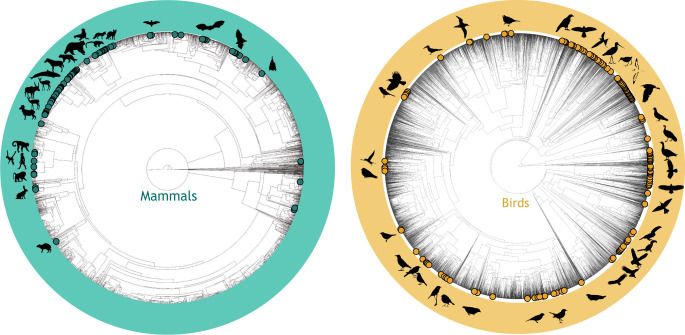
**Mammal and bird phylogenetic representation of 240 genera for which both tracking and trait data are available (dots) relative to all species (grey lines).** Based on the *D*-statistic (Fritz and Purvis, 2010), there is extensive phylogenetic clustering of available trait and tracking data (*D*=0.797 for mammals, *D*=0.657 for birds). For mammals, we used the phylogeny from Upham et al. (2019); for birds, we used the phylogeny from Jetz et al. (2008). Representative *phylopic* silhouettes are shown for a subset of 173 genera.

**Fig. 2.**
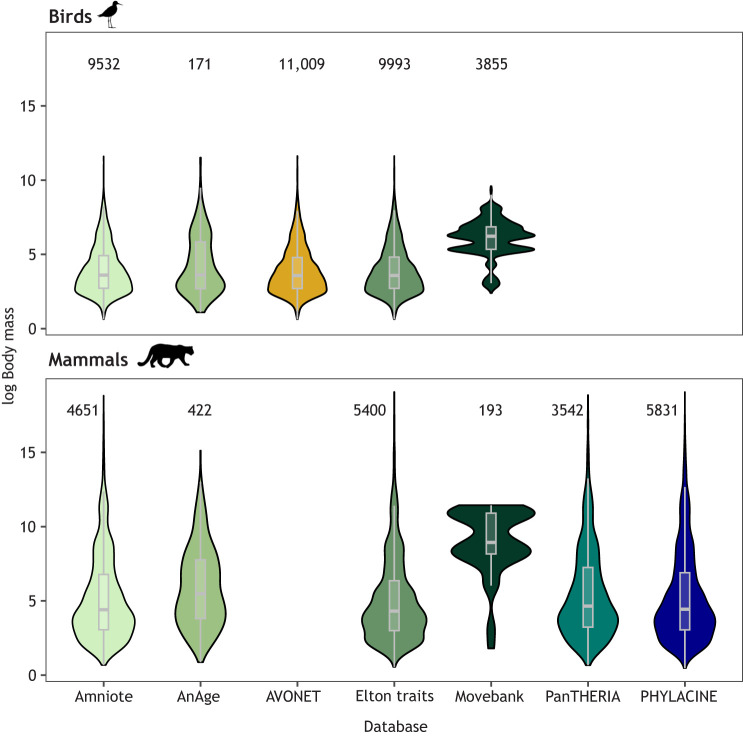
**Publicly available trait and tracking datasets overlap in their representation of animal size (body mass) but the Movebank database tends to include larger animals within each Class.** Body mass (in g) was log transformed. Numbers above the violin plots represent the count of individual animals for each Class in each database. Please note that some databases only contain data from one Class (AVONET: birds, PanTHERIA and PHYLACINE: mammals) and therefore no body size data are shown.

There are data for many more species in trait databases than in tracking databases. Trait databases contain data for thousands of species (AVONET: 11,009 bird species, Amniote: 14,183, AnAge: 593, COMBINE: 6234, EltonTraits: 15,393, PHYLACINE: 5831, PanTHERIA: 3542) whereas tracking databases include fewer species (e.g. Movebank: 1400 species). Across all trait databases, data are available for 19,662 species of which only 1.8% (362 species) also have publicly available tracking data. This low overlap means that trait-tracking integration efforts are currently possible for a remarkably low percentage of the tree of life, thus greatly hindering our current potential for generality in macroecology. These 362 species (265 bird species and 96 mammal species) are from 240 genera and 35 orders (Fig. 1; Figs S1 and S2). Clearly, the integration of trait and tracking databases will inevitably be constrained by the number of species with movement data available in tracking databases. However, trait databases do not have dense representation for all the species that are most abundant in tracking databases, such as the largest mammals, and tracking data sometimes come from a biased subset of populations. Asymmetric availability of trait data (Etard et al., 2020), tracking data (Hazen et al., 2012) and demographic data (Conde et al., 2019) is well established. Given the limitations in the size of tracking instruments and the important ethical considerations associated with tracking instrument size relative to animal size (Payne et al., 2024 preprint), taxa in tracking databases are generally larger bodied than those represented in trait databases (Fig. 2). However, there is substantial overlap in both size and species representation, which makes integration of trait and tracking databases possible, and new methods for automated monitoring of smaller animals in the field might further close this gap (Böhner et al., 2023).

### Disparities across geography and time

**Fig. 3.**
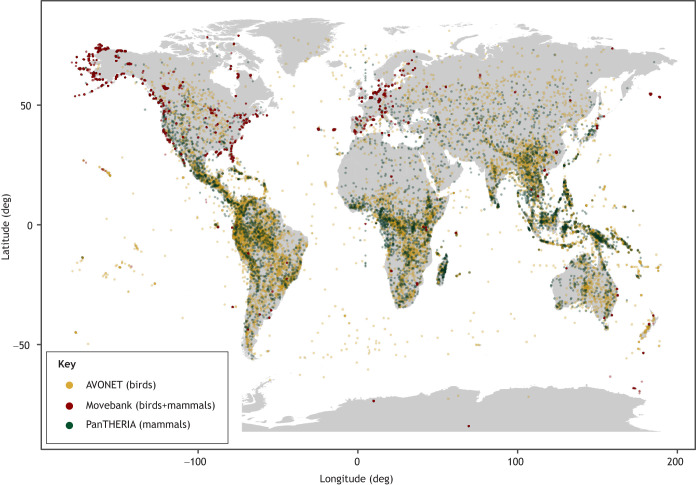
**Geographic distribution of publicly available data from trait and tracking databases.** Whereas trait data (AVONET and PanTHERIA) are globally distributed, reflecting increased species richness near the tropics, publicly available tracking data (Movebank) are predominantly collected in the USA and Europe. Note that tracking database points are deployment locations (i.e. 1 point per deployment), not recovery locations or tracking data. Data are only shown for the 33% of available studies (163 studies with 9640 deployments) that provided deployment location data.

Trait and tracking data collection are often limited to a small portion of species ranges. As a result, species-specific trait data are often pieced together from several geographically distant sources (e.g. adult body mass from one continent, limb length, brain size or age at maturity from another continent, etc.) or from several sources that lack georeferenced data. This approach obscures potentially important geographical variation and trade-offs. Moreover, a substantial fraction of long-term animal ecology datasets represent areas of the planet with low biodiversity (Titley et al., 2017) or with limited vulnerability to climate change (Salguero-Gómez et al., 2021; Paniw et al., 2021). Therefore, piecing together traits and tracking data from geographically or taxonomically biased sources can lead to incomplete or even erroneous conclusions (Trimble and van Aarde, 2012). Likewise, information about when the trait measurements or specimens were collected is increasingly important for studies about biotic responses to environmental change. Climate change is dramatically shaping traits and movement patterns in animals. For example, some birds are becoming smaller (Weeks et al., 2020), some migrations are being threatened (Gross, 2024) and some geographic distributions are shifting (Martins et al., 2024). Thus, it may be problematic to integrate historic trait measurements with contemporary tracking data [e.g. those from AVONET collected in the 1970s, when few animals were instrumented with biologgers because of technological limitations (Kooyman, 2004)]. When integrating trait and tracking databases, researchers should note when and where measurements were made. To quantitatively assess geographic distributions of trait and tracking measurements, we created a map of metadata from the three databases that contained geographic location data (centroid latitude/longitude for AVONET, mid-range latitude/longitude for PanTHERIA, and deployment latitude/longitude for Movebank) (Fig. 3). We found that available tracking data deployment locations are predominantly from the USA and Europe, whereas trait data are much more widely available overall, and reflect the much higher species diversity in Central and South America, Africa and Asia. Gaps in both datasets reflect broader geographic biases in research coverage, including physiological metrics (White et al., 2021) and conservation research (Reboredo Segovia et al., 2020). Similar to the data-sharing disparities (see ‘Disparities in data-sharing norms’, above), the disparities in geographic distribution of tracking databases may be due to the large cost associated with tracking studies. However, note that tracking data locations have a broader geographic distribution than deployment locations (Kays et al., 2020).

### Disconnections across disciplines

Why are tracking and trait data not integrated more often? We argue that this disconnect is the result of disciplinary silos including publication venues rather than a reflection of its potential. Trait databases are often used to address evolutionary or community ecology questions, whereas tracking data are often used to address questions related to behavioral ecology or applied wildlife management and conservation. To test this hypothesis, we searched for papers citing the foundational publication of four trait databases and one movement database in Web of Science. We used the *refsplitr* package in R (Fournier et al., 2020) to parse the references by journal name, and tallied the number of times each database publication was referenced in each journal (Fig. S3). We also examined a measure of impact in each of the journals, the h5-index value from Google Scholar, which represents the h-index for articles published in the last 5 complete years (i.e. the largest number h such that h articles published in 2018–2022 have at least h citations each). We found that the foundational Movebank publication (Kranstauber et al., 2011) tends to be cited in different journals (e.g. Ecology and Evolution, Ecography, Journal of Animal Ecology) which have lower impact factors (h5-index range 61–67) as compared with the foundational trait database publications [PHYLACINE (Faurby et al., 2018), PanTHERIA (Jones et al., 2009), COMBINE (Soria et al., 2021), AVONET (Tobias et al., 2022), AnAge (De Magalhães and Costa, 2009)], which tend to be cited in interdisciplinary journals such as Proceedings of the Royal Society B and PLOS ONE with higher impact factors (h5-index range 83–212). This is not an insurmountable problem, but requires proactive efforts to break down disciplinary silos.

## Case studies

Overlap in data availability suggests that integrating trait and tracking databases is possible. Here, we provide three ideas for case studies to demonstrate the potential utility of integration. Together, these three case studies show that integrating trait and tracking databases is possible and shows great promise.

### Metabolics case study

**Fig. 4.**
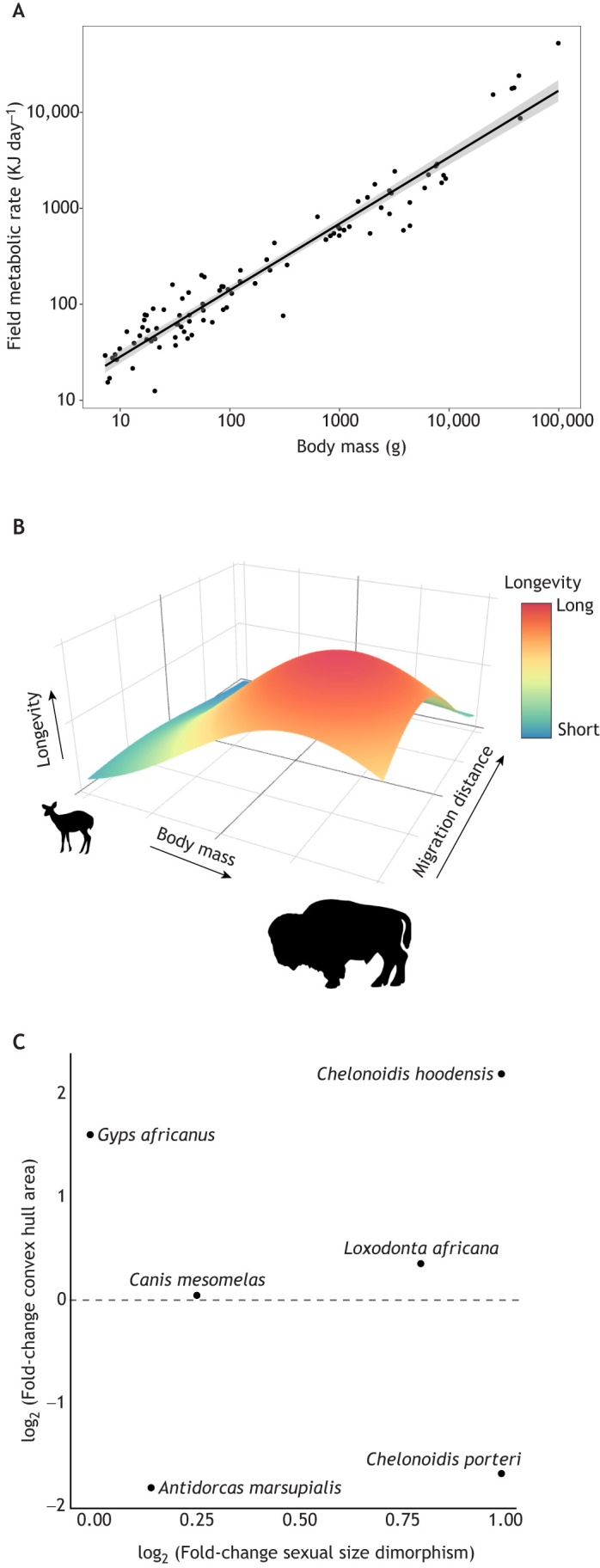
**Three case studies showing the potential utility of integrating trait and tracking databases.** (A) Metabolics case study: field metabolic rate plotted against body mass for 95 species of eutherian and marsupial mammals (data from Nagy et al., 1999). Integration of trait and tracking data (e.g. migratory status, daily movement distance) could be used to explain deviations from this line. (B) Migration distance case study: a hypothetical relationship between body size, migration distance and longevity for herbivores. Available tracking and trait data could be used to test this hypothesized relationship. (C) Sexual niche segregation case study: data from trait and tracking data combined to demonstrate how their integration could be used to examine whether sexual size dimorphism is related to differences in movement patterns. The *x*-axis shows the log_2_ of the ratio of male:female mass (positive numbers show males having larger body masses than females) and the *y*-axis shows the log_2_ of the ratio of male:female home range convex hull area (positive numbers show males having larger home range sizes than females).

In birds, computer simulations suggest that the relationship between behavior, environment and life history is fundamentally mediated by metabolism (Pierce et al., 2024). However, disconnections between biomechanics, bioenergetics and ecology have resulted in major unanswered questions about how the energetic costs of locomotion scale up to entire migrations. One important question in ecology is: how much does movement explain deviations between field metabolic rate and body mass? Theory has shown that the costs of migration are extensive, but nuanced energetic and demographic trade-offs exist (Brown et al., 2023). Trait databases (McNab, 2008; Herberstein et al., 2022) contain an enormous amount of field metabolic rate and body mass data at the species level (Fig. 4A). Variation around the relationship between field metabolic rate and body mass may be explained with many metrics derived from tracking data. For example, longer migration distances or increased daily movement distance could contribute to both heightened basal and field metabolic costs. Theory could be used as a starting point to determine which tracking and trait metrics are ripe for integration.

### Size-specific longevity case study

Ungulates vary in body mass from the approximately 1.5 kg Java mouse deer (*Tragulus javanicus*) to the approximately 1500 kg hippopotamus (*Hippopotamus amphibius*). Nearly half of all extant ungulate species migrate (Abraham et al., 2022) and some larger bodied ungulates undertake extremely long migrations. For example, caribou (*Rangifer tarandus*) have one of the longest migrations of any terrestrial mammal (Joly et al., 2019). The evolution of longer migrations facilitated larger body size (Abraham et al., 2022); however, support for whether or not large body size facilitates longer migrations is inconsistent (Joly et al., 2019; Teitelbaum et al., 2015). In contrast, larger body size is suggested to increase longevity because of the ‘live slow, die old’ life history strategy that typifies most large species (Gaillard et al., 2003; Lindstedt and Calder, 1981). Although large body size increases migration distance and longevity, trade-offs exist between migration distance and longevity as migrations can increase survivorship by allowing individuals to exploit productive foraging grounds but can also reduce survival as a result of factors such as energetic constraints of travel and risk of predation or disturbances (Sawyer et al., 2019; Passoni et al., 2021; Kentie et al., 2023). Thus, understanding the interactions between body size, migration distance and longevity will help disentangle some of the driving forces behind macroecological patterns and elucidate trade-offs between migration distance and longevity. Integration of existing species-level tracking and trait data could be used to ask: can migration distance help explain some of the variance in the longevity–body size relationship, and can body size influence the migration distance–longevity relationship? Potential hypotheses include: (H_0_) migration distance is unrelated to longevity and (H_1_) animals that migrate further have shorter longevity when controlling for body size. Researchers could analyze individual-level tracking data [e.g. from Joly et al. (2019) or Movebank or Euromammals] along with species-level size and longevity data from other databases [e.g. from AnAge (De Magalhães and Costa, 2009)] to identify macroecological patterns across herbivores (Fig. 4B) and other mammals. Currently, body size (Smith et al., 2003), longevity (Myhrvold et al., 2015; De Magalhães and Costa, 2009) and migration distance (Joly et al., 2019) data exist for 11 ungulate species; however, expansion of this dataset will increase statistical power and allow for more robust conclusions to be made. The analysis could also be applied to birds, which experience most of their mortality during migrations (Sillett and Holmes, 2002) but seem to have higher annual adult survival with increased migration distance (Winger and Pegan, 2021).

### Sexual niche divergence case study

Sexual size dimorphism and sexual niche partitioning are widespread phenomena amongst vertebrates (Tombak et al., 2024), and are thoroughly studied in ungulates (Ruckstuhl and Neuhaus, 2000). Hypothesized proximate and ultimate causes of sexual niche partitioning include differential predation risk, foraging strategies and activity budgets. These hypotheses can be mechanistically tested by combining biologging to quantify space use, movement behavior and activity patterns with trait measurements such as sex, size and metabolic rates at the individual level. Biologgers have been attached to males and females in many species separately (Breed et al., 2006), but large comparative analyses have yet to be done. For these questions, individual-based tracking data and trait measurements are needed because they capture intraspecific variation, especially in metrics such as migration distance and home range stability that often vary according to ‘movement syndromes’ (such as residents, migrants and nomads) that exist within and between species (Kays et al., 2023). The combination of tracking and trait data should allow for broader syntheses to elucidate the causes and consequences of sexual segregation.

As a proof of concept, we downloaded a multi-species tracking dataset from the Movebank Data Repository (https://doi.org/10.5441/001/1.hm5nk220; see Abrahms et al., 2017), retaining tracks from species where both adult males and females were instrumented, yielding six species: one bird (*Gyps africanus*), three mammals (*Antidorcas marsupialis*, *Canis mesomelas*, and *Loxodonta africana*), and two reptiles (*Chelonoidis hoodensis* and *Chelonoidis porteri*). The sexual size dimorphism of these six species ranges from the monomorphic *G. africanus* to the 2:1 male:female size ratio of *Chelonoidis* (Mundy et al., 1992; Skinner and Chimimba, 2005; Bonin et al., 2006; Laws et al., 1975). We tested whether sexual size dimorphism was associated with space use using a simple approach: (1) estimating space use by each individual as the area of the convex hull containing their track (minimum 90 days) and (2) calculating the log fold-change of the mean convex hull area between males and females (Fig. 4C). We could increase analytical power with larger sample sizes and more sophisticated estimates of home range size. However, major challenges for this approach are data access limitations and heterogeneity in data characteristics and structure (e.g. sampling rates, location accuracy), which demand substantial effort to synthesize tracking studies for comparison with trait databases.

## Integration into the future

We are entering a new and data-rich era of possibilities for comparative work (Salguero-Gómez et al., 2021). The transition of scientific journals from print to online publication has revolutionized the use of supplemental data and living databases (Odlyzko, 2002), and accelerated momentum toward open science (Ramachandran et al., 2021). It is now increasingly possible to combine trait and tracking databases to discover broad patterns and make predictions, even about species whose traits or movement patterns cannot yet be measured at scale. Future integration efforts can be accelerated in four ways. First, higher discoverability and more open access to tracking data will be critical for expanding the number and types of taxa in comparative studies. Rapid technological innovations including Motus and ICARUS are making it possible to instrument progressively smaller animals and understudied taxa such as ectotherms, which will increase the promise of trait–tracking integration in the future, but only if the data are openly accessible and taxonomically and spatiotemporally indexed. Second, future research, especially synthesis efforts, will benefit from measurements of more and different individual-level traits. For example, physiological, bioenergetic and biomechanical traits are nearly absent from trait databases (e.g. muscle fiber anatomy, reaction speed, maneuverability, fuel-carrying capacity, stride length, cost of transport, body temperature ranges, fat stores). These mechanistic underpinnings of animal movement are critically important for understanding ecology, evolution and conservation, including for making predictions about animal persistence in our changing world. Measuring multiple traits per individual (e.g. from museum specimens and biologged individuals) can fill in gaps for living individuals where few traits are measured. Coordinated networks across scales of biological organization could be used to measure linked traits (Culina et al., 2021). Third, researchers across both domains need to make individual-level trait data available. As noted above, the majority of trait databases have historically reported data at the species level, despite the importance of intraspecific variation (Des Roches et al., 2018). The impact of within-species variability (Violle et al., 2007) on ecological and evolutionary processes can be important even if means are the same (Weller et al., 2024). Finally, future comparative efforts will benefit from integration with other types of databases not discussed here, including population models [e.g. matrix population models through COMADRE (Salguero-Gómez et al., 2016), PADRINO (Levin et al., 2022) and MOSAIC (Bernard et al., 2023)], other movement data (e.g. camera trap and ringing/banding/tagging data), environmental data (e.g. remote sensing) and genomic data.

## Conclusion

Data on animal traits and movement patterns have independently provided invaluable insights because they each represent a critical link between phenotypic and behavioral characteristics of organisms and their roles in populations, communities and ecosystems (Guralnick et al., 2016). We believe that harmonization of trait and tracking databases would facilitate major exploration and discovery, including quantifying and predicting animal responses to climate change and landscape fragmentation, explaining performance limits and understanding demographic drivers. Integration of trait and tracking data with other types of databases would also allow us to reflect on sources of bias in our understanding of the natural world (e.g. how representative are population model databases or tracking databases in terms of biomes, foraging strata and nocturnality versus diurnality). This integration will require our research communities to continue collection and deposition efforts strategically and openly.

Major remaining opportunities exist to improve geographic, taxonomic and disciplinary overlap between trait datasets and tracking datasets, especially at the individual level. A key challenge is the inevitable trade-off between nuance and generalization in grouping data; species have been used as sampling units for macroecological research (Tobias et al., 2022) because variance in trait values among species usually outweighs within-species variance (Pigot et al., 2020; but see Des Roches et al., 2018). Numerous opportunities also exist to leverage artificial intelligence to bridge the traits–tracking gap, including by training artificial intelligence to estimate traits of tracked individuals, either from images such as camera trap photos or from regression-based approaches trained from other datasets. As the pace of climate change accelerates, data-driven conservation efforts using species traits and tracking patterns are needed (Jetz et al., 2019; Guralnick, 2017). We argue that the expansion of publicly available animal tracking and trait databases makes now an ideal time to combine the two into a rich quantitative framework for developing and testing hypotheses in vertebrate animals across geographic, temporal and biological allometric scales.

## Supplementary Material

10.1242/jexbio.247981_sup1 Supplementary information

Table S2. Table of the 616 publicly available animal tracking studies from Movebank as of April 2024. Each study is referenced as a DOI for the dataset where available, or else as a published paper or report cited in the study where available, and otherwise by the Movebank study name. A study can be viewed by appending the study ID to the following URL: https://www.movebank.org/cms/webapp?gwt_fragment=page=studies,path=study.
