# Supplementary material for: Integrating animal tracking and trait data to facilitate global ecological discoveries
Source: J Exp Biol. 2025 Feb 20;228(Suppl 1):JEB247981. doi: 10.1242/jeb.247981 (PMC11883293; doi:10.1242/jeb.247981)
Supplement: Supplementary information [file jexbio-228-247981-s1.pdf]

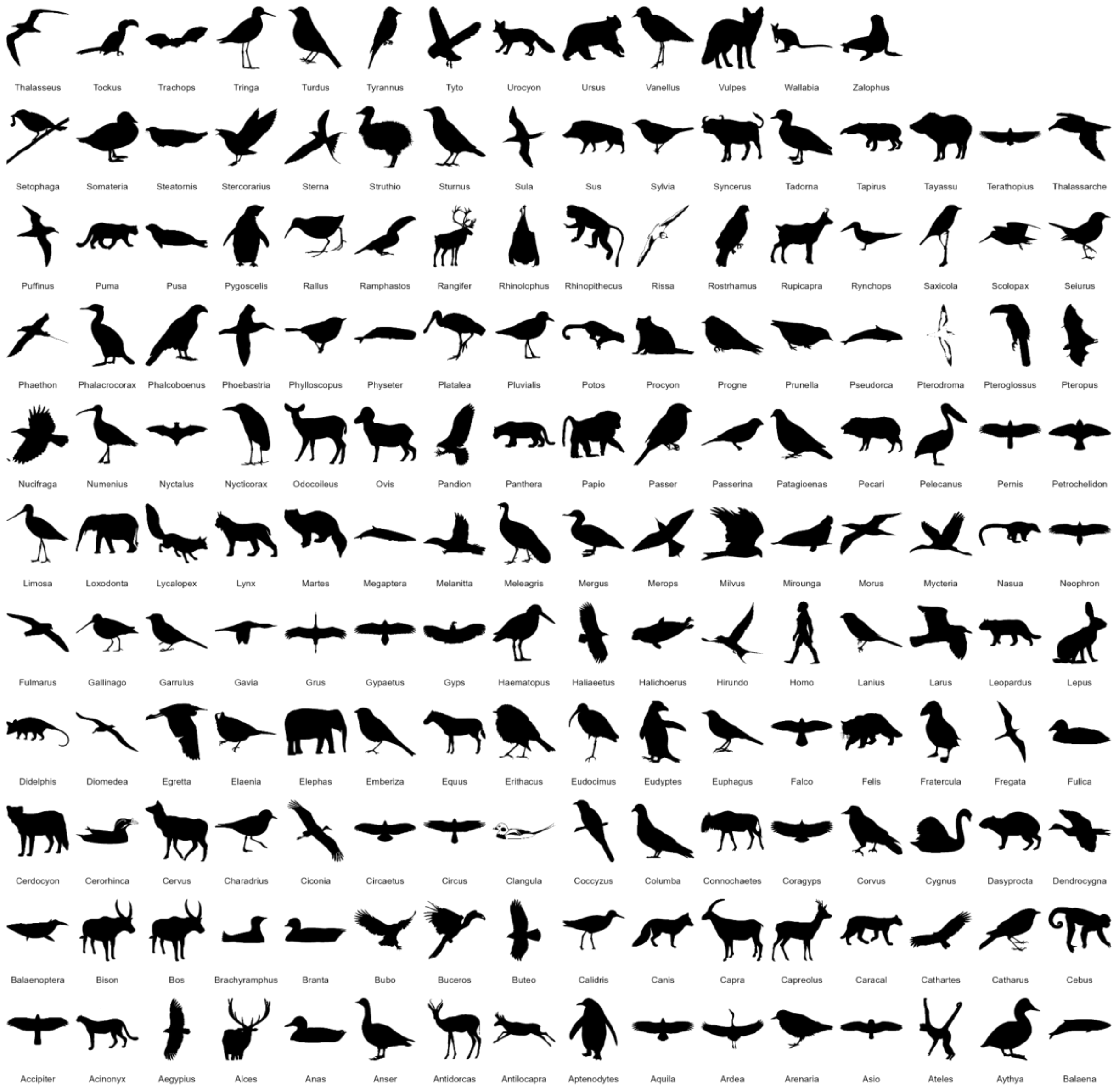

Fig. S1. Birds and mammals from 240 genera have both tracking and trait data available. A subset of 173 genera with available *phylopic* silhouettes are shown here in alphabetical order by genus.

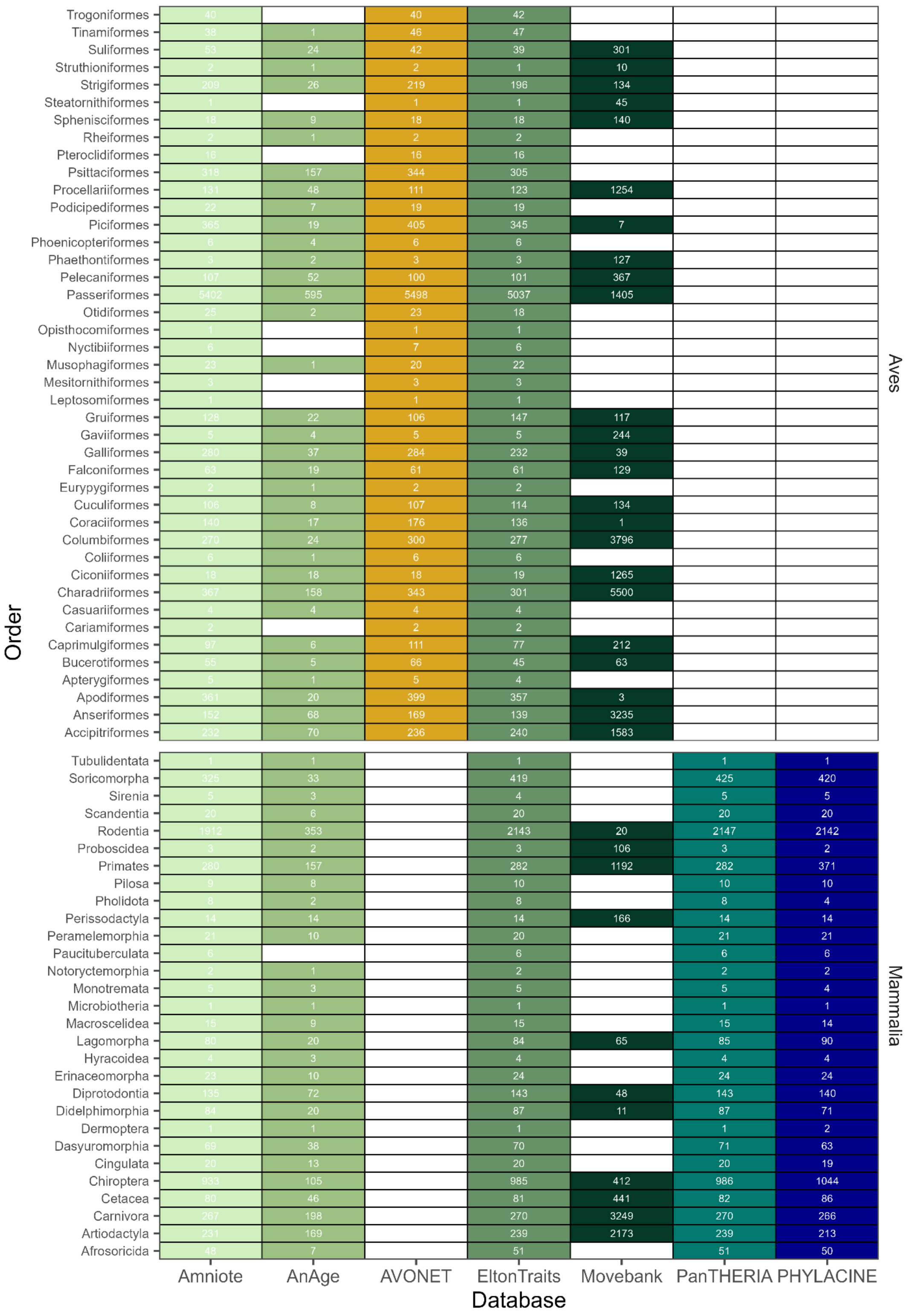

Fig. S2. Orders represented by each database, using the *taxizedb* package in R to assign each genus and species to an order (based on ITIS). Numbers represent the count of individual animals from each order in each database. Note that all orders have

Journal of Experimental Biology • Supplementary information

trait data available (from Amniote and EltonTraits databases) but not all orders have tracking data available (from the Movebank database). Likewise, some databases only contain data from one class (AVONET = birds, PanTHERIA and PHYLACINE = mammals) and therefore no count data are shown.

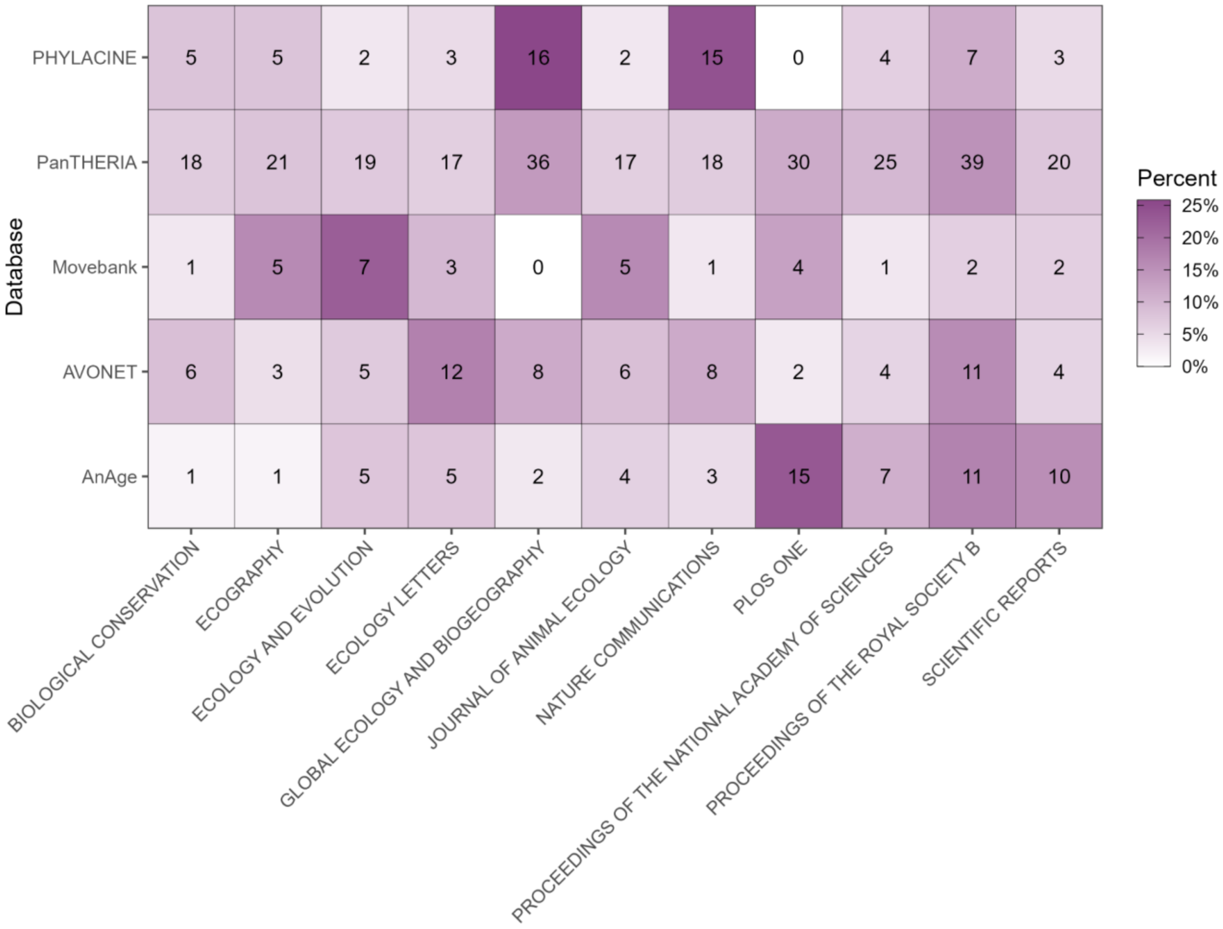

Fig. S3. Numbers of peer-reviewed publications that cite the foundational trait and tracking database papers are published in various journals. Only journals with >30 citations across the foundational publications are shown.

Journal of Experimental Biology • Supplementary information

Table S1. Detailed descriptions of the seven databases featured in this Commentary.

Database Name	Database Type	Data Description	Organisms	Taxonomic Level	Spatial Extent	Reference
MoveBank	Tracking	Animal tracking data collected using animal-borne sensors	All	Individual	Global	https://besjournals.onlinelibrary.wiley.com/doi/full/10.1111/2041-210X.13767
Amniote	Trait	Life history traits including longevity, reproductive traits, and body size	Birds, Mammals, Reptiles	Species	Global	https://esajournals.onlinelibrary.wiley.com/doi/abs/10.1890/15-0846R.1
AnAge	Trait	Longevity records, life history traits, reproductive traits, physiological traits	Vertebrates	Species	Global	https://doi.org/10.1111/j.1420-9101.2009.01783.x
AVONET	Trait	Functional trait data including ecological variables and morphological traits	Birds	Individual, Species	Global	https://onlinelibrary.wiley.com/doi/full/10.1111/ele.13898
EltonTraits	Trait	Diet, foraging stratum, activity, body mass	Mammals and Birds	Species	Global	https://doi.org/10.1890/13-1917.1

Journal of Experimental Biology • Supplementary information

PHYLACINE	Trait	Range maps, trait data, and threat status	Mammals	Species	Global	https://esajournals.onlinelibrary.wiley.com/doi/10.1002/ecy.2443
PanTHERIA	Trait	Life history, ecology, geography	Mammals	Species	Global	https://esajournals.onlinelibrary.wiley.com/doi/epdf/10.1890/08-1494.1

Table S2. Table of the 616 publicly available animal tracking studies from Movebank as of April 2024. Each study is referenced as a DOI for the dataset where available, or else as a published paper or report cited in the study where available, and otherwise by the Movebank study name. A study can be viewed by appending the study ID to the following URL: https://www.movebank.org/cms/webapp?gwt_fragment=page=studies,path=study .

Available for download at
https://journals.biologists.com/jeb/article-lookup/doi/10.1242/jeb.247981#supplementary-data

Journal of Experimental Biology • Supplementary information
